# Defining *Plasmodium falciparum* Treatment in South West Asia: A Randomized Trial Comparing Artesunate or Primaquine Combined with Chloroquine or SP

**DOI:** 10.1371/journal.pone.0028957

**Published:** 2012-01-31

**Authors:** Kate Kolaczinski, Toby Leslie, Iftikhar Ali, Naeem Durrani, Sue Lee, Marion Barends, Khalid Beshir, Rosalynn Ord, Rachel Hallett, Mark Rowland

**Affiliations:** 1 London School of Hygiene and Tropical Medicine, London, United Kingdom; 2 HealthNet TPO, Peshawar, Pakistan; 3 Faculty of Tropical Medicine, Mahidol University, Bangkok, Thailand; 4 Centre for Clinical Vaccinology and Tropical Medicine, Churchill Hospital, Oxford, United Kingdom; 5 Shoklo Malaria Research Unit, Mae Sot, Thailand; Walter & Eliza Hall Institute, Australia

## Abstract

**Introduction:**

Antimalarial resistance has led to a global policy of artemisinin-based combination therapy. Despite growing resistance chloroquine (CQ) remained until recently the official first-line treatment for falciparum malaria in Pakistan, with sulfadoxine-pyrimethamine (SP) second-line. Co-treatment with the gametocytocidal primaquine (PQ) is recommended for transmission control in South Asia. The relative effect of artesunate (AS) or primaquine, as partner drugs, on clinical outcomes and gametocyte carriage in this setting were unknown.

**Methods:**

A single-blinded, randomized trial among Afghan refugees in Pakistan compared six treatment arms: CQ; CQ+(single-dose)PQ; CQ+(3 d)AS; SP; SP+(single-dose)PQ, and SP+(3 d)AS. The objectives were to compare treatment failure rates and effect on gametocyte carriage, of CQ or SP monotherapy against the respective combinations (PQ or AS). Outcomes included trophozoite and gametocyte clearance (read by light microscopy), and clinical and parasitological failure.

**Findings:**

A total of 308 (87%) patients completed the trial. Failure rates by day 28 were: CQ 55/68 (81%); CQ+AS 19/67 (28%), SP 4/41 (9.8%), SP+AS 1/41 (2.4%). The addition of PQ to CQ or SP did not affect failure rates (CQ+PQ 49/67 (73%) failed; SP+PQ 5/33 (16%) failed). AS was superior to PQ at clearing gametocytes; gametocytes were seen on d7 in 85% of CQ, 40% of CQ+PQ, 21% of CQ+AS, 91% of SP, 76% of SP+PQ and 23% of SP+AS treated patients. PQ was more effective at clearing older gametocyte infections whereas AS was more effective at preventing emergence of mature gametocytes, except in cases that recrudesced.

**Conclusions:**

CQ is no longer appropriate by itself or in combination. These findings influenced the replacement of CQ with SP+AS for first-line treatment of uncomplicated falciparum malaria in the WHO Eastern Mediterranean Region. The threat of SP resistance remains as SP monotherapy is still common. Three day AS was superior to single-dose PQ for reducing gametocyte carriage.

**Trial Registration:**

**ClinicalTrials.gov** bold>

## Introduction

Antimalarial drug resistance is an ongoing threat to malaria control [1]. Despite widespread documented resistance, chloroquine remained widely used in Pakistan and Afghanistan for first line treatment of falciparum malaria at the time of this study (2000–2003) [2]–[4]. In many other parts of the world including Pakistan and Afghanistan the antifolate drug sulfadoxine-pyrimethamine (SP) is comparatively effective against falciparum malaria in contrast to the situation in South East Asia and many African settings. In Pakistan and Afghanistan transmission is seasonal, strong immunity seldom develops, infected individuals are symptomatic and it is thought that the majority seek treatment [2], [3], [5]. These conditions expose the majority of infections to antimalarial drugs and would be expected to exert strong selection for resistance [6]. SP has a long plasma half-life which may further contribute to selection. In such areas of low seasonal transmission the operational effectiveness of SP before resistance arises appears to be short [7]. Whilst current effectiveness remains comparatively good in the region low level *in vivo* resistance to SP has been demonstrated [5], [8], [9]. Wide-scale adoption of effective SP based combinations, rather than SP monotherapy, which was being considered as an option to replace chloroquine at the time of the study, could delay the selection of resistance and prolong the useful life of SP across the subcontinent.

In areas of low or medium endemicity co-treatment of infections with a gametocytocidal drug may help to reduce transmission and may be particularly important when considering strategies for malaria elimination. In South Asia, the standard policy has been to co-treat with a single dose of primaquine to reduce gametocyte carriage. Primaquine is known to have poor efficacy as a direct treatment but has repeatedly been shown to be highly gametocytocidal [10]–[14]. Reduction in gametocyte carriage and infectivity to mosquitoes after artesunate treatment is widely documented [15], [16]. It appears to be due to a combination of rapid clearance of asexual stages, direct activity on immature gametocytes (either killing them or preventing their maturation) and possibly reduction in the infectivity of mature gametocytes [17], [18]. Use of ACTs has been shown to reduce transmission [19]–[23] and is recommended for epidemic response [24], as too is primaquine [25]. There is increasing interest in using primaquine with ACTs to accelerate gametocyte clearance [14]. SP treatment of clinical infections can lead to high gametocyte loads which persist for 10 days or more [15] with latent gametocytaemia detected by PCR for up to a year [26].

A direct comparison of artesunate and primaquine on treatment failure and gametocyte carriage has not been conducted before. Many Afghan refugee communities in Pakistan have a history of falciparum malaria and are prone to outbreaks [27]–[29]. A trial examining the comparative efficacy of chloroquine and SP combined with either artesunate or primaquine was therefore carried out in refugee villages in North-western Pakistan.

## Materials and Methods

*The protocol for this trial and supporting CONSORT checklist are available as supporting information; see Checklist S1 and Protocol S1.*

### Study area and population

The study recruited patients from five Afghan refugee villages within an 80 km radius of Peshawar, Khyber Province (formerly North West Frontier Province), Pakistan. The 5 villages were established in the early 1980s and each has a history of falciparum transmission. Three (Adizai, Naguman and Yakka Ghund) are situated on the banks of the Kabul river, and two (Mohammed Khoja and Kotki) are situated to the south of Peshawar in Kohat district. Malaria transmission is seasonal with vivax malaria occurring from March to November and falciparum from July to December.

Participants were Afghan refugees who were permanently resident in the Pakistan villages. A minority might have acquired their infections in Afghanistan, but admission criteria required 4–6 weeks of follow-up which would have excluded the more regular travellers.

### Study sites

The study was conducted through three sites with well-managed clinics run by Non-Governmental Organisations (NGO) and government agencies. Site 1 was Adizai village which also served the population of nearby Naguman. Site 2 was Yakka Ghund village. Site 3 was Kotki which also serviced Mohammed Khoja village. Health staff from the NGO HealthNet TPO were seconded to each clinic to manage the recruitment and follow-up of patients.

The study took place over three malaria seasons from 2000 to 2003. In the first season only site 1 was used, in the second season site 2 was added, and in the third season site 3 was added. The stepped inclusion of sites resulted from unexpectedly low recruitment rates at the first study site, and is summarized in Table 1. An earlier *in vivo* survey across all refugee camps along the length of the 1000 km North-South axis of Khyber Province showed little heterogeneity in resistance frequency to chloroquine and SP [30]. (Note that SP is the only drug name abbreviated throughout the text. Abbreviations for chloroquine (CQ), primaquine (PQ) and artesunate (AS) are used when referring to specific study arms (e.g. CQ+PQ) but not at other points in the text.)

**Table 1:** Summary of treatment arms tested at each of the study sites over the three transmission seasons.

	Study site		
Transmission season (July–January)	Site 1 (Adizai)	Site 2 (Yakka Ghund)	Site 3 (Kotki)
Season 1: 2000–2001	CQ, CQ+PQ, CQ+AS,SP, SP+PQ, SP+AS		
Season 2: 2001–2002	SP, SP+PQ, SP+AS	CQ, CQ+PQ, CQ+AS	
Season 3: 2002–2003	CQ, CQ+PQ, CQ+AS	SP, SP+PQ, SP+AS	CQ, CQ+PQ, CQ+AS

***Study design and procedures.*** Individuals presenting with clinical symptoms and diagnosed microscopically with falciparum malaria were referred to trial staff for further assessment. Inclusion criteria were: over two years of age, not pregnant, *P. falciparum* mono-infection, more than 1 asexual parasite per 10 fields, no other serious disease, resident in the refugee village for the full period of follow-up, no verbal report of antimalarial use during the last 21 days, and no signs of severe malaria.

Individuals meeting the inclusion criteria and giving informed consent were allocated using randomization tables stratified by age and sex to the following treatment arms: chloroquine (CQ^1^) (25 mg/kg) over 3 days; CQ (25 mg/kg) over 3 days plus primaquine (PQ) (0.5 mg/kg) on the last day of treatment; CQ (25 mg/kg) over 3 days plus artesunate (AS) (4 mg/kg per day) over 3 days; sulfadoxine (25 mg/kg) and pyrimethamine (1.25 mg/kg) (SP) on day 0; SP (25:1.25 mg/kg) plus PQ (0.5 mg/kg) on day 0; SP (25:1.25 mg/kg) on day 0 plus AS (4 mg/kg per day) over 3 days. The primaquine co-treatment regimens were based on recommendations for use of primaquine as a gametocytocide treatment for falciparum malaria [31]. On treatment days when any specific arm did not require a partner drug, a placebo was substituted.

Patients were not allocated to all six treatment arms at each site (Table 1). During years two and three, at any one site, patients were allocated to *either* one of the three SP treatment arms *or* one of the three chloroquine treatment arms; only in year one were patients allocated to all six treatment arms in the original site.

For each of the study sites patients were stratified by age and sex and assigned consecutive patient numbers at enrolment. Treatment groups were pre-assigned to patient numbers using simple randomization lists generated using Excel (Microsoft Corp., Seattle, USA). The assignment of treatment group to patient number was concealed until after enrolment. The random allocation sequence was generated by two of the investigators (KK, MR) neither of whom enrolled or assessed patients.

The brands and manufacturers/suppliers were: SP (Fansidar **®**, Roche), chloroquine (Nivaquine **®**, Beacon), primaquine (IDA), artesunate (Plasmotrim **™**, Mepha). Although chloroquine resistance was known to exist in the study area, chloroquine was the first line treatment policy of the Government of Pakistan and the UN refugee agency (UNHCR) at the time and was therefore included.

The trial manager, who allocated the treatment arms and administered treatment, was not blind to the study drugs and allocations. Recruited patients, microscopists and health workers were partially blinded; full blinding was not achieved given (i) the appearance of the different drugs, and (ii) the different times of follow up for the SP arms compared to the chloroquine arms.

Patients were given directly observed treatment (by the trial manager), monitored for 30 minutes and re-dosed if vomiting occurred. Health workers then recorded the other day 0 biomedical parameters.

Patients were asked to return on each day of treatment (days 0, 1, 2) and on days 3, 7, 14, 21 and 28 post treatment. Patients in the three SP arms were also followed up on days 35 and 42 for detection of late recrudescence [32]. On each day of follow up thick and thin smears were taken, clinical symptoms recorded and blood spots collected on Whatman No. 1 filter paper for parasite typing and differentiation of recrudescent from new infections. Haematocrits and blood spots were taken either on the day of failure or on day 28. Patients not presenting at the clinic were followed up at home. Criteria for withdrawal were reported administration of additional anti-malarial drugs (protocol violation), emergence of any concomitant febrile illness that interfered with outcome classification (including a non-falciparum malaria, for which the withdrawn study patient would be given appropriate first line treatment), parasitaemia still present on day 7, or signs of severe malaria developing before completing the initial treatment course.

Patients found to be parasitaemic on any day after day 3 were treated with SP (the official second line treatment) or with SP and mefloquine (Fansimef, Roche) for those whose initial treatment was SP based. SP resistant parasites were sensitive to the mefloquine component of Fansimef, which was used *in lieu* of mefloquine monotherapy which was unavailable in the study area. Those who developed severe malaria, severe anaemia or other complications were referred to Khyber Hospital, Peshawar for treatment.

**Laboratory tests.** Thick and thin blood smears were stained with 2% Giemsa solution. All slides were read on the day of collection by a microscopist based at each site. Differential diagnosis of vivax and falciparum (trophozoites and gametocytes) was by examination of thick and thin smears according to standard microscopy methods [33]. Trophozoites and gametocytes were counted against 200 white blood cells (WBC) from the thick blood smear on the assumption of a WBC count of 8000/µl. A smear was declared negative if no parasites were seen after examining 100 fields. Slides from sites 2 and 3 were re-examined for accuracy by the site 1 microscopist. Comparison was made between the parasite counts made by the three microscopists. The mean variation in parasite count for trophozoites and gametocytes was less than 5%. Owing to electricity supply deficiency, the haematocrit microcentrifuge was only used at sites 1 and 3.

**Outcome measures.** Patients completed the trial if treatment was administered fully and all follow-up appointments conducted, or if they failed treatment on any day of follow-up. The primary endpoint of the trial was any clinical or parasitological failure up to day 28, although a subset of patients in the SP treatment arms was also followed until day 42. Patient outcomes were classified under the WHO parasitological classification system of sensitive (S) or resistant (RI, RII, RIII) infections with those whose outcomes were classified as S (sensitive) being treatment successes and those with outcomes of RI, RII or RIII being classified as treatment failures [34]. This classification system was still commonly used at the time of the study and the ethical clearance for this study (received in 2000) was based on a protocol using this classification system. Patients were also classified against the newer WHO treatment outcome system [35], [36]. Presenting the outcomes under both of these systems allows better comparability with data past and present [37]. For the WHO classification system standard definitions were applied: adequate clinical and parasitological response (ACPR), early treatment failure (ETF) or late treatment failure (LTF) which incorporated standard definitions of late clinical and parasitological response [35]. Those cases with ETF or LTF were classified as treatment failures, and ACPR was classified as treatment success if they completed the follow-up period.

Other parameters examined were time to fever resolution (with fever defined as axillary temperature ≥37.5°C), asexual parasite clearance, gametocyte clearance and gametocyte carriage on or after day 7.

**Molecular characterisation.** PCR genotyping was conducted on a subset of samples to distinguish recrudescent from new infections using the protocol described by Brockman et al [38]. *P. falciparum genes msp-1*, *msp-2* and *glurp* were genotyped according to polymorphisms present at variable loci on day 0 and day of failure. Approximately 50% of cases were available for genotyping.

PCR corrected outcomes were applied as a secondary analysis using redefined outcomes based on the number and frequency of true recrudescent infections and reclassifying cases with new infections as treatment successes, excluding those with indeterminate outcomes or negative PCR results. PCR testing was performed at the Shoklo Malaria Research Unit, Thailand.

We also conducted an analysis at the London School of Hygiene and Tropical Medicine (LSHTM), UK, for known resistance-associated mutations to chloroquine and antifolate drugs. Samples collected at enrolment were tested for known mutations in the *pfcrt*, *pfmdr1*, *pfdhfr* and pf*dhps* genes [39], [40]. PCR and sequence specific oligonucleotide probe assays were used to analyse nucleotide polymorphisms at *pfcrt codon 76*, *pfmdr1* codons 86 and 184, *pfdhfr* codons 16, 51, 59 and 108 and *pfdhps* codons 436, 437, 581 and 613 [41].

### Sample size and data analysis

The sample size required to detect a difference in treatment failure or gametocyte carriage with 80% power and 95% confidence (two-sided significance level for type 1 error of 0.05) were calculated using the following predictions. In the chloroquine arms (i) the estimated frequency of failure in chloroquine monotherapy arm was 30% and in each of the combination arms (CQ+PQ or CQ+AS) it was 10% or less; (ii) the estimated proportion of gametocyte positive individuals after 7 days in the chloroquine arm was 50% and in each of the combination arms it was 25%. In the SP arms (i) the estimated frequency of failure in the SP arm was 10% and in each of the combination arms (SP+PQ or SP+AS) it was 1%; (ii) the estimated frequency of gametocyte positive patients after 7 days in the SP arm was 50% and in each combination arm it was 25%. The estimated samples sizes were 64 per arm in the chloroquine arms and 121 per arm in the SP arms. For gametocyte carriage the required sample sizes were 65 per arm. The target sample sizes for the study were 65 for the chloroquine arms and 121 for the SP arms. To allow for 15% loss to follow up the targets for recruitment were 76 for the chloroquine arms and 142 for the SP arms.

The primary aims of the study were to evaluate: 1) the relative efficacy of the combination drugs in providing parasite clearance with no recrudescence compared to monotherapy, and 2) the effect on gametocyte clearance. The secondary aim was to determine the proportion of individuals in each arm with classifiable treatment failure.

The primary outcome was the proportion of individuals in each treatment group classified as a treatment failure during the 28 days follow-up compared to the respective monotherapy. The odds of treatment failure between each of the study groups at day 28 were estimated after adjusting for potential confounders (age, sex, parasitaemia, PCV, study site) using logistic regression analysis or Mantel-Haenszel χ^2^ test. Treatment failure was also analysed using the definitions of ACPR and ETF or LTF, and parasitological classifications using the S-R scale in addition to the PCR corrected analysis [35]. Time to event data (time to treatment failure) were analysed with Kaplan Meier survival analysis.

For assessing potential effects on gametocyte carriage, the primary outcome was the presence or absence of gametocytes on day 7 with a secondary analysis examining the presence or absence of gametocytes and the geometric mean gametocyte density on each day of follow-up.

All data were entered in Microsoft Excel (1997) by one data clerk and the database checked patient by patient against paper records by a second data clerk. Analysis was performed using STATA 10.0 (STATA Corp, College Station, TX, USA).

### Ethics Statement

The study protocol was approved by the ethics committees of the Pakistan Medical Research Council and LSHTM, UK. All patients or their guardians gave written informed consent to participate. The trial was registered under clinicaltrials.gov [NCT00959517].

## Results

### Recruitment and follow-up

**Figure 1:**
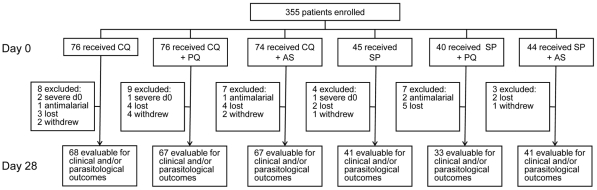
Trial profile.

A total of 355 cases of microscopically confirmed falciparum malaria were enrolled into the study between July 2000 and December 2002. Table 2 shows enrolment characteristics and Figure 1 shows the trial profile. Patients were recruited to the chloroquine arms in line with recruitment targets. Insufficient numbers were recruited to the SP arms. This was due to considerably lower than expected malaria cases occurring in these locations during the study period (in contrast to the years leading up to the study). Additional sites were added over the three-year study period to increase recruitment but it was not possible to continue the trial for longer because of resource constraints. chloroquine arms met the recruitment targets because of the site allocations and unexpected pattern of case loads in the third year.

**Table 2:** Enrolment characteristics of the treatment groups.

Variable	CQ	CQ+PQ	CQ+AS	SP	SP+PQ	SP+AS
Number enrolled	76	76	74	45	40	44
Number evaluable at day 28 (%)	68 (89)	67 (88)	67 (91)	41 (91)	33 (82)	41 (93)
Age [median (IQR) years]	12 (8–18)	12 (8–20)	12 (8–20)	17 (9–27)	14 (7–25)	18.5 (9.5–30)
Percentage female	42	37	50	33	43	45
Weight [median (IQR) kg]	29 (20–47)	30 (20–53)	33 (20–45)	48 (25–58)	42 (22–55)	41 (21–57)
Temperature [mean (SD) °C]	37.3 (1.0)	37.5 (1.2)	37.4 (1.2)	37.5 (1.0)	37.5 (1.2)	37.5 (1.5)
Temperature ≥37.5°C on presentation [n (%)]	33 (43)	34 (45)	34 (46)	21 (47)	17 (43)	23 (52)
PCV [mean (SD) % haematocrit]^1^	42.9 (9.7)	40.8 (3.9)	41.5 (4.3)	44.2 (7.7)	45.5 (6.9)	44.3 (5.1)
PCV<30% [n (%)]^1^	0	0	0	1 (2.2)	0	0
Asexual parasite density [geometric mean (95% CI) per µl]	5161 (3536–7535)	5263 (3647–7595)	7366 (4972–10,915)	7600 (5185–11,140)	8091 (4236–15,454)	12,134 (7757–18,982)
Gametocyte positive [n (%)]	12 (15.8)	15 (19.7)	9 (12.2)	14 (31.1)	7 (17.5)	7 (15.9)
Gametocyte density [geometric mean (95% CI) per µl]	1.3 (0.4–2.6)	1.7 (0.7–3.4)	0.6 (0.2–1.2)	4.1 (1.4–10.1)	1.6 (0.3–4.1)	1.2 (0.2–2.9)

Notes: (1) PCV was not recorded for all patients; a microcentrifuge was only available at one of the 3 clinics. For PCV percentages in the 6 treatment groups: CQ n = 10; CQ+PQ n = 19; CQ+AS n = 13; SP n = 19; SP+PQ n = 15, SP+AS n = 15.

Characteristics of patients recruited into the 3 chloroquine arms were broadly similar, as were the three SP arms. The SP+AS arm showed slightly higher asexual parasite densities on enrolment than other groups (Table 2).

Of 355 patients enrolled, 38 (10.7%) were either withdrawn or lost to follow up leaving 317 for possible inclusion (Figure 1). Of these 292 (82%) were evaluable for parasitological outcomes because 25 patients classified as RII parasitological failure did not meet the definition of clinical failure, i.e. they did not have fever on day 3. Likewise, 308 (87%) were evaluable for clinical outcomes because 9 patients who had classifiable Early Treatment Failure did not fit the definition for parasitological failures (they did not reach day 7 of the trial). Of the 266 patients followed up for 42 days, 221 (83%) were evaluable for clinical and parasitological outcomes. All treatments were well tolerated and no severe or serious adverse events were recorded during the study.

**Figure 2:**
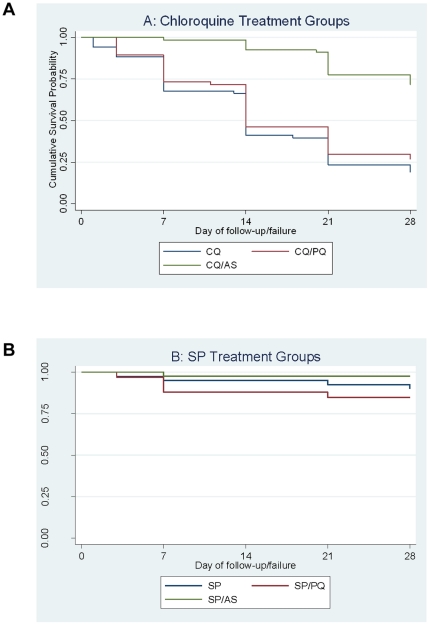
Kaplan Meier survival analysis showing cumulative probability of failure in CQ treatment groups (A) and SP treatment groups (B).

**Clinical Outcomes.** The 28-day failure rate in the CQ and CQ+PQ arms was 81% and 73% respectively (Table 3, Figure 2A). The addition of artesunate improved the treatment response, with a treatment failure rate of 27%. The failure rates in the SP groups were 16% or less (Table 3) with the combination of SP+AS having the lowest failure rate (1/41 [2.4%]). Neither SP+AS nor SP+PQ arms were significantly different to the SP arm at the 28-day end point (Figure 2B).

**Table 3:** Clinical and parasitological failure rates at 28 days follow-up and for gametocytaemia at day 7.

	Day 28 clinical or parasitological failure			Day 7 gametocytaemia		
	No./Total (%)	Odds Ratio (95% CI)	P	No./Total (%)	Odds Ratio (95% CI)	P
CQ	55/68 (81)	Reference	-	52/67 (78)	Reference	-
CQ+PQ	49/67 (73)	0.64 (0.29–1.4)	0.3	27/70 (39)	0.18 (0.09–0.4)	<0.001
CQ+AS	19/67 (28)	0.09 (0.04–0.2)	<0.001	12/72 (17)	0.06 (0.02–0.1)	<0.001
SP	4/41 (10)	Reference	-	37/43 (86.1)	Reference	-
SP+PQ	5/33 (16)	1.6 (0.41–6.7)	0.5	24/36 (66.7)	0.32 (0.10–1.0)	0.046
SP+AS	1/41 (2)	0.23 (0.02–2.2)	0.2	9/44 (20.5)	0.04 (0.01–0.1)	<0.001

None of the potential confounding factors examined (age, sex, parasitaemia, study site, PCV) were associated with treatment outcome so adjustments were unnecessary in the final regression analysis. Crude odds ratios are presented.

Clinical and parasitological outcomes are shown in Table 4. Only 32/133 (24%) of the individuals who failed had fever on the day of failure; the majority of ‘late treatment failures’ therefore fell into the category of ‘late parasitological failure’. Some recent history of fever may have gone unreported.

**Table 4:** Clinical and parasitological outcomes after 28 days follow-up: n (%).

	CQ	CQ+PQ	CQ+AS	SP	SP+PQ	SP+AS
Clinical outcomes	N = 56	N = 58	N = 67	N = 41	N = 30	N = 40
Adequate clinical response	13 (23)	18 (31)	48 (72)	37 (90)	28 (93)	40 (100)
Early treatment failure	9 (16)	7 (12)	0	1 (2)	1 (3)	0
Late clinical failure	2 (4)	4 (7)	1 (2)	0	0	0
Late Parasitological Failure	32 (57)	35 (50)	18 (26)	3 (7)	1 (3)	0
Parasitological outcomes	N = 63	N = 65	N = 67	N = 40	N = 32	N = 41
S	13 (21)	18 (28)	48 (72)	37 (93)	28 (88)	40 (98)
RI	35 (56)	33 (51)	19 (28)	3 (7)	1 (3)	0
RII	13 (21)	11 (16.9)	0	0	3 (9)	1 (2)
RIII	2 (3)	3 (5)	0	0	0	0

Trophozoite clearance time (days until negative smear) was lowest for the artesunate combination arms. Addition of primaquine to either chloroquine or SP did not appear to affect clearance times. Clearance times for each arm (median (interquartile range)) were: CQ, 3 days (2–7); CQ+PQ, 3 days (2–7); CQ+AS, 2 days (1–2); SP, 2 days (2–3); SP+PQ, 2 days (1.5–3); SP+AS, 1 day (1–2).

Of the 133 failures, 79 (47%) matched pairs were available for PCR evaluation of MSP-1, MSP-2 and GLURP. Matched pairs were collected on day 0 and on the day of failure. Outcomes of the PCR are shown in Table 5, which excludes those with indeterminate results. Most re-infections took place at one site (Site 3, Yakka Ghund) where malaria transmission was higher than in the other villages. Treatment outcomes were reclassified for those failures where matched pairs were available and used to give adjusted failure rates for the sample (Table 5). Although the number of failures in the chloroquine arms was reduced by the correction, the failure rates remained high at >50% for chloroquine monotherapy. The PCR adjusted treatment failure rate of the CQ+AS combination (9%) was considerably lower that the in vivo rate (28%).

**Table 5:** Outcomes of PCR analysis, and projected adjusted failure rates by treatment group excluding indeterminate results.

	PCR Results [n (%)]				Failure rates [n/N (%)]	
	Reinfection	Recrudescent	Negative	Total	*in vivo* ^1^	PCR adjusted ^2^
CQ	6 (21)	20 (69)	3 (10)	29	55/68 (81)	42/68 (62)
CQ+PQ	4 (21)	11 (58)	4 (21)	19	49/67 (73)	36/67 (54)
CQ+AS	7 (64)	3 (27)	1 (9)	11	19/67 (28)	6/67 (9)
SP	0	2 (100)	0	2	4/41 (10)	4/41 (10)
SP+PQ	0	2 (100)	0	2	5/33 (16)	5/33 (16)
SP+AS	-	-	-	0	1/41 (2)	1/41 (2)

Notes: (1) Taken from Table 3 for 28 day failures; (2) Adjusted by the ratio of PCR recrudescent to PCR re-infected cases to estimate number of true recrudescent cases among the observed *in vivo* failures.

In the subset of patients followed-up for 42 days, recrudescence between day 28 and 42 was rare: only 8/317 (2.5%) of individuals classified as adequate clinical and parasitological response (ACPR) or sensitive (S) at day 28 went on to fail by day 42 (1 in the CQ arm, 2 in the CQ+PQ arm, 3 in the CQ+AS arm, 1 in the SP arm and 1 in the SP+PQ arm). Failure rates at day 42 were therefore similar to day 28. The failures between 28-day and 42-day rates were not corrected by PCR and hence could have resulted from new infections.

**Molecular outcomes.** Molecular analysis of drug resistance-associated alleles was conducted on samples taken on day 0 (Table 6). The major marker of chloroquine resistance, *pfcrt*-76T, was fixed at 100%. Mutations in p*fmdr1* are known to modulate resistance to quinoline and other drugs. The prevalence of chloroquine-resistance associated allele *pfmdr1*-86Y was 13.6% in this sample. Mutations associated with pyrimethamine resistance (on the *pfdhfr* gene) were seen frequently; 108 N was found in all but one of 74 typable samples (98.6%) and 59R occurred in 66/76 (86.8%) samples. However, 51I was only detected in 5/75 (6.7%) samples. Therefore *pfdhfr* ‘double mutants’ were common (at codons 108+59) but ‘triple mutants’ were rare. Interestingly, we observed one sample with the *pfdhfr* mutations 16V+108T. These are reportedly selected by the antifolate drug cycloguanil, rather than pyrimethamine [42]. No mutations on the *dhps* gene were seen. These PCR results broadly match what would be expected from the clinical outcomes. The *pfdhr/pfdhps* mutation profile suggests that the parasites would be killed by a full therapeutic dose of SP, but that some tolerance to lower doses existed.

**Table 6:** Drug resistance alleles in a sub-set collected at enrolment.

Locus	Allele	Number	(%)
Pfcrt	76 K	0	
	76 T	63	(100)
Pfmdr1	86 N	76	(86)
	86 Y	12	(14)
	184 Y	22	(27)
	184 F	60	(73)
DHFR	16 A	76	(99)
	16 V	1	(1)
	50/51 CN1	70	(93)
	50/51 C1	5	(7)
	59 C	10	(13)
	59 R	66	(67)
	108 N	73	(99)
	108 T	1	(1)
DHPS	436/437 SA	43	(100)
	581 A	41	(100)
	613 A	43	(100)

### Effect on Gametocytes

The addition of artesunate or primaquine to chloroquine or SP reduced gametocyte carriage on day 7 (Table 3). Combining chloroquine or SP with artesunate succeeded in eliminating gametocytaemia from more individuals by day 7 compared to monotherapy or co-treatment with a single-dose of primaquine.

**Figure 3:**
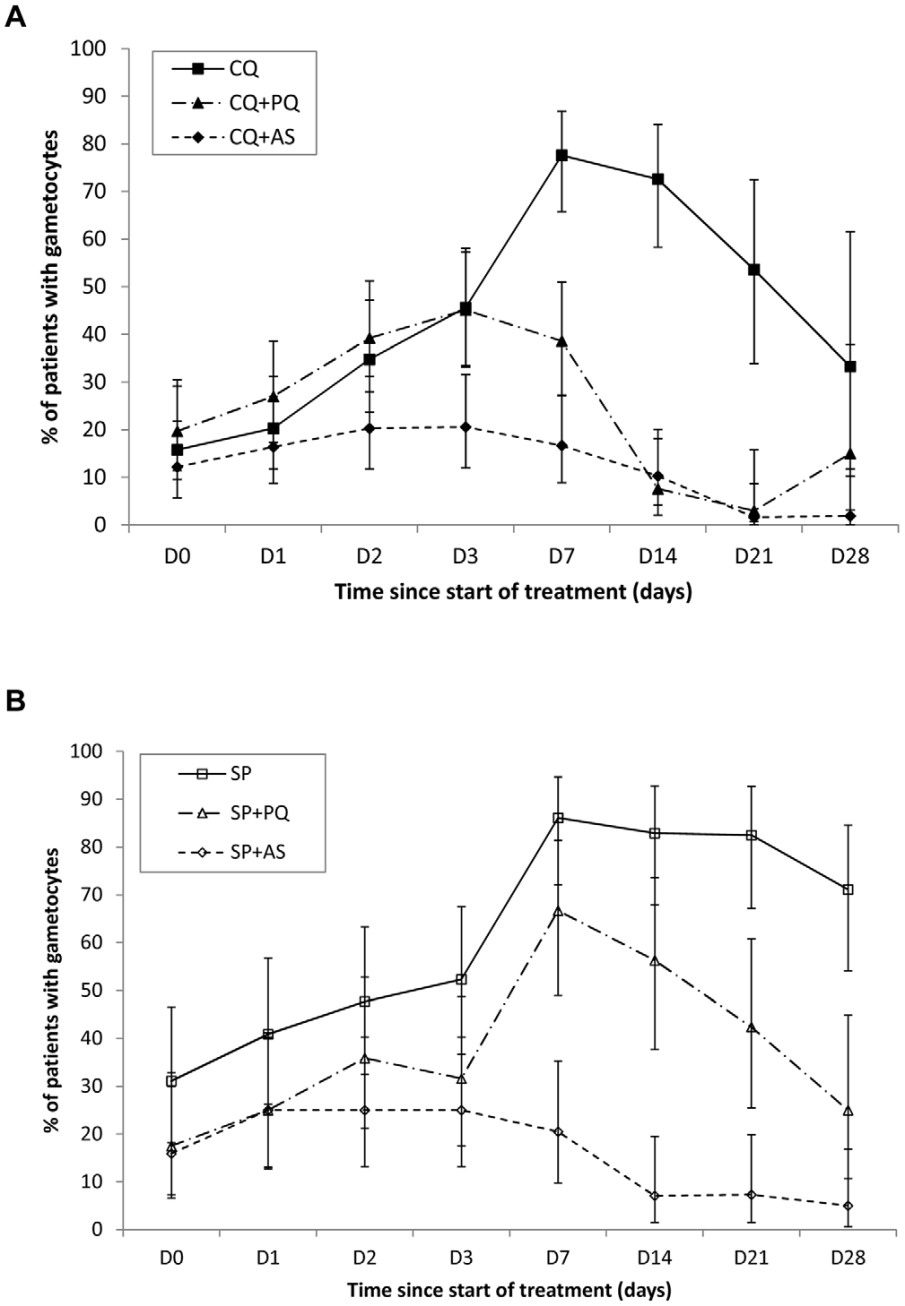
Percentage of patients (+CI) carrying gametocytes on specified days after treatment in CQ (A) and SP (B) treatment arms.

**Figure 4:**
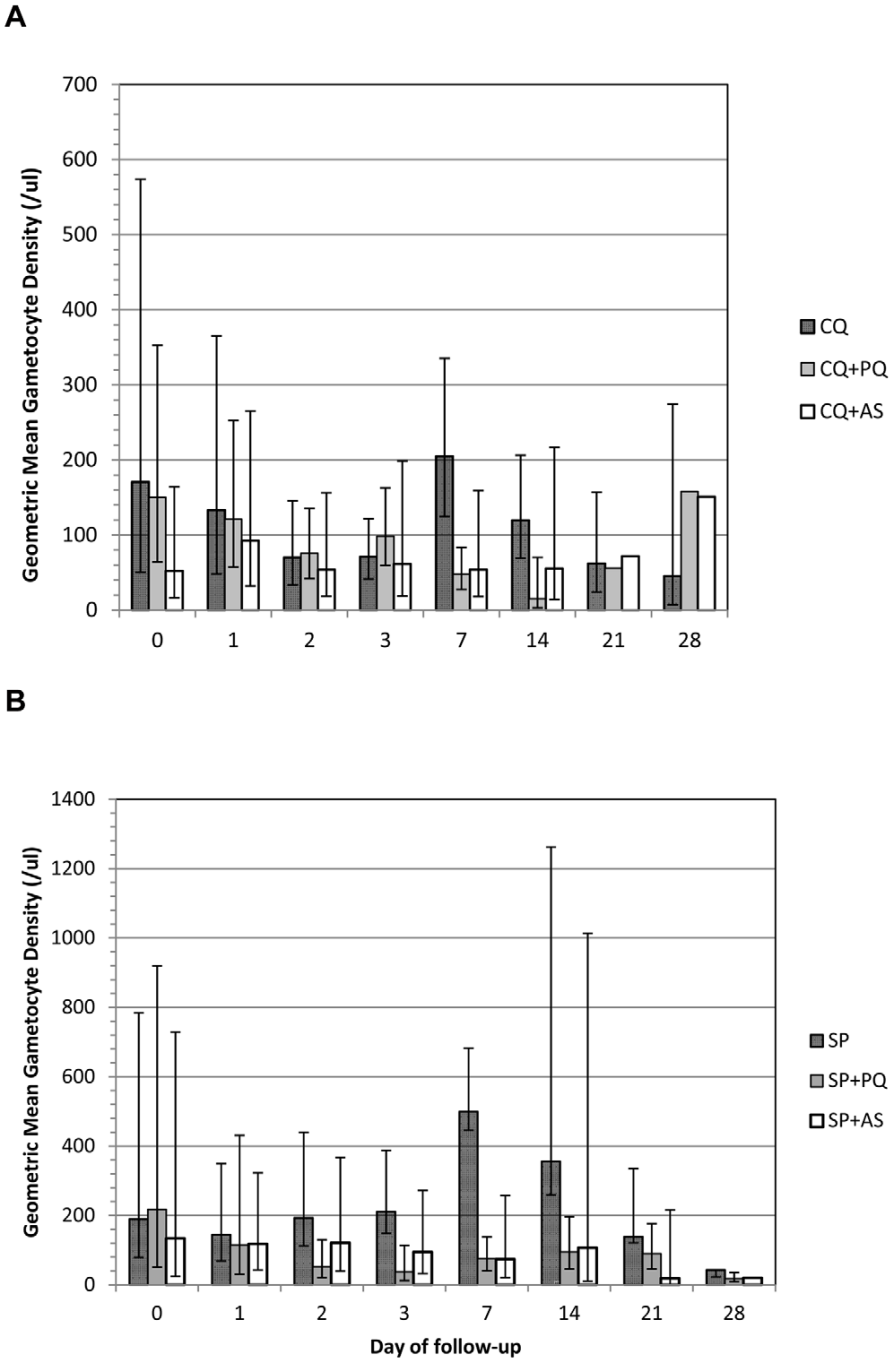
Geometric mean gametocyte density (+CI) after treatment in chloroquine (A) and SP treatment arms (B). Note the different scales on the Y-axis. Where the CI bars are absent, only 1 patient had gametocytes.

Figure 3 shows the proportion of individuals with patent gametocytaemia over 28 days of follow-up. In the chloroquine and SP arms, gametocyte carriage persisted at day 28 in >30% of individuals in the CQ arm and >70% of individuals in the SP arm. The addition of primaquine reduced gametocyte carriage; the effect was more pronounced with chloroquine than with SP. The proportion of gametocytaemic individuals was lowest in the artesunate arms and the difference was evident within two days of the start of treatment. Peak gametocyte densities occurred 7 days after the start of treatment (Figure 4). Gametocyte density was higher after SP than after chloroquine treatment. Both primaquine and artesunate reduced density to low levels by day 7.

Most patients did not have gametocytaemia on day 0 (291/355 (82.0%) had no gametocytes on day 0). However, the presence or absence of sexual stage parasites on day 0 was an important explanatory variable in the secondary analysis; patients who presented with gametocytes on day 0 were more likely to be gametocytaemic on day 7 than individuals who were not gametocytaemic on day 0 regardless of treatment group (Mantel-Haenszel OR: 3.5 [95%CI: 2.0–6.4], p<0.001). We therefore conducted further analysis comparing those who presented with gametocytes on day 0 and those who did not.

The artesunate treatments were more effective at preventing the emergence of gametocytaemia between day 0 and 7 than in removing established gametocytaemia already present on day 0 (Table 7). For example, among the CQ+AS group who *did have* patent gametocytaemia on day 0, 8/9 were still gametocytaemic on day 7 after treatment. This was not significantly different from the proportion gametocytaemic on day 7 (11/12) after treatment with chloroquine monotherapy (Fisher's exact test p = 1.0). By contrast, in the chloroquine arms of the patients who *did not have* patent gametocytaemia on day 0, only 11% (7/63) were gametocytaemic on day 7 when treated with CQ+AS as compared to 47/56 (84%) among those treated with chloroquine monotherapy (χ^2^ p<0.001). Similar trends were evident in the SP arms treated with artesunate; prevalence of gametocytaemia on day 7 was significantly lower in the SP+AS group that *was not* gametocytaemic on day 0 than in the group that *was* (Fisher's exact test p<0.001). This indicates that artesunate is less active against older gametocytes than against those newly emerged or immature forms that are not yet emerged. Primaquine showed no such trend: the proportion of patients gametocytaemic on day 7 was not significantly different for patients who *were* or *were not* gametocytaemic on day 0. Primaquine therefore appears to be active against gametocytes of all ages.

**Table 7:** Numbers and percentages of individuals with gametocytes on day 7 post treatment.

	No (%) parasitaemic on day 7	
	Amongst those who had gametocytes on day 0	Amongst those who did not have gametocytes on day 0
CQ	11/12 (91.7%) ^a, 1^	47/56 (83.9%) ^a, 1^
CQ+PQ	7/14 (50%) ^b, 1^	21/56 (37.5%) ^b, 1^
CQ+AS	8/9(88.9%) ^a, 1^	7/63(11.1%) ^c, 2^
SP	14/14 (100%) ^a, 1^	25/29 (86.2%) ^a, 1^
SP+PQ	5/7 (71.4%) ^b, 1^	23/30 (76.7%) ^a, 1^
SP+AS	5/7 (71.4%) ^b, 1^	5/37 (13.5) ^b, 2^

Chloroquine or SP treatments in the same column that share the same superscript letter are not significantly different. Treatments in the same row that share the same numeric superscript are not significantly different.

Primaquine was more effective at controlling gametocytaemia when combined with CQ than with SP, regardless of whether individuals were gametocytaemic at the start of treatment (MH-OR = 0.22, P = 0.001) (Table 7). By contrast, artesunate seemed as effective when combined with chloroquine as it was when combined with SP (MH-OR = 1.04, P = 0.83). Though, as discussed, artesunate was more effective at preventing development of patent gametocytaemia than in clearing existing gametocytaemia. Artesunate was better than primaquine at preventing patent gametocytaemia among those that were not gametocytaemic at day 0 enrolment (CQ: OR = 0.30, P<0.001; SP: OR = 0.18, P<0.001).

Using the presence of gametocytes on day 7 as the secondary endpoint, adjusted for the presence of gametocytes at day 0, both primaquine and artesunate with chloroquine or SP were more effective than their respective monotherapies at reducing the presence of gametocytes (Table 8).

**Table 8:** Effect of treatment on gametocyte carriage at day 7 and day 28, for those patients who were gametocytaemic on enrolment.

	AOR for presence of gametocytes on day 7, (95% CI), p-value ^1^	AOR for presence of gametocytes on day 28, (95% CI), p-value ^1^
CQ	Reference	Reference
CQ+PQ	0.15 (0.07–0.34), p<0.001	0.42 (0.08–2.2), p = 0.3
CQ+AS	0.05 (0.02–0.12), p<0.001	0.04 (0.004–0.39), p = 0.006
SP	Reference	Reference
SP+PQ	0.37 (0.12–1.16), p = 0.09	0.14 (0.05–0.4), p<0.001
SP+AS	0.04 (0.01–0.13), p<0.001	0.02 (0.005–0.1), p<0.001

Notes: (1) AOR: Odds ratios using logistic regression analysis adjusted for presence of gametocytes on day 0 (CQ and SP arms analysed separately).

Compared to monotherapy with chloroquine or SP, co-treatment with artesunate was negatively associated with having gametocytes on day 28 whether the accompanying drug was chloroquine or SP (Table 8). The presence of gametocytes on day 0 was not associated with the presence of gametocytes on day 28 (MH-OR, adjusted for treatment arm: 1.0, p = 1.0) suggesting that older gametocytes had cleared by day 28, regardless of treatment.

## Discussion

The present study was designed to inform decision-making at several levels: to guide UNHCR on appropriate treatment in the Afghan refugee communities in Pakistan, to build evidence for national treatment policy in the Pakistan Directorate of Malaria Control and in the Afghanistan Ministry of Public Health, to guide the WHO Eastern Mediterranean Region Office (EMRO) on regional treatment recommendations, and to build evidence for international epidemic response guidelines. The findings of this study, when presented at an inter-country meeting of national malaria control managers coordinated by WHO/EMRO in 2004, contributed to a shift in policy from chloroquine monotherapy to ACT in South and West Asia [43], influencing the defining of SP+AS as the first line treatment of choice for the WHO Eastern Mediterranean Region. India is the latest neighbouring country to adopt SP+AS as first line treatment with the support of WHO [37]. The SP+AS combination is also effective against vivax malaria [44], although not as an approved treatment.

SP monotherapy was effective in the study population, with treatment failure rates remaining in the 5–10% range, similar to that seen in other studies in the region [5], [8], [45]. Molecular studies in the region show that genetic markers for SP resistance are still at low levels in the parasite population [46], [47]. However, the South East Asian experience of rapid rise in SP resistance in settings of similar endemicity [7] points to SP resistance spreading rapidly if ACT policy is not implemented rigorously in both public and private sectors across the region [37].

The complete failure of chloroquine (which at the time of the study remained the officially sanctioned first line treatment) convinced policy makers of the need to redefine treatment practices in South Asia. The new data demonstrated higher levels of resistance than in previous studies dating back to the 1980s and 1990s [2], [3], [5], [48], [49] and emphasised the need for change to national treatment guidelines. The addition of artesunate to chloroquine did reduce treatment failure rates, but if this regimen was used as policy it would, in effect, constitute artesunate monotherapy in the majority of infections and accelerate the development of artesunate tolerance, as reported in South East Asia [50].

A constraint on the study design was that not all 6 arms could be included at each of the three study sites. Site is therefore a potential confounding covariate that cannot be fully controlled for in the data analysis. Differences in transmission could, for example, affect the risk of re-infection between sites but this was corrected for by the PCR analysis. At each site in any one year each triplet of treatment arms (all the SP arms or all the chloroquine arms) were examined at the same time, and it is comparisons within these triplets which are of greatest interest. The study did not complete its target sample size for the SP arms because of low recruitment rates.

PCR corrected outcomes were not attainable for all failures, and higher numbers of patients were reinfected at one site where only the chloroquine arms were being tested. Low rates of transmission at the other sites make it unlikely that failing cases were due to reinfection. The secondary analysis with PCR-corrected outcomes did not change the overall conclusions on the suitability of the combinations for revised treatment policy.

Treatment with either the clinically effective SP monotherapy or the failing chloroquine monotherapy resulted in persistence of gametocytaemia into the second and third weeks in the majority of individuals. This effect was more evident for SP than for chloroquine, SP being well known for high rates of gametocyte carriage post treatment [32]. This contrasts with situations of low-frequency chloroquine resistance where the proportion of gametocytaemic patients post treatment also tends to be lower [16], [51].

The effect of artemisinin derivatives on reducing gametocyte carriage is already documented [16], [52]. Artesunate also proved highly effective in reducing the prevalence of gametocytaemia in the present study. Regimens combining artesunate with chloroquine or SP saw marked reductions in gametocyte carriage and few cases had persisting gametocytaemia. As demonstrated with SP+AS in The Gambia [16] the SP+AS and CQ+AS combinations appear to have limited activity against mature circulating gametocytes [53]. Co-treatment with a single dose of primaquine was more effective against older gametocyte infections. Both primaquine and, more markedly, artesunate reduced the odds of persisting gametocytes from that seen post treatment with either chloroquine or SP monotherapy.

Primaquine is more rapidly excreted than artesunate and this may account for the fact that the single dose of primaquine used in this study had a lower impact on gametocyte carriage than the artesunate regimens. The usefulness of primaquine as a gametocytocidal treatment may be improved by administering it after the ACT course [14], [54].

In our study, a key factor in the clearance of gametocytes was the presence or absence of gametocytes on day 0. Those who presented with gametocytes were more likely to have gametocytes on day 7, an effect which was independent of the treatment given. Although the majority of patients (80%) presented without gametocytaemia, artesunate appeared to be less active against these older parasites whereas primaquine appeared to be effective against all ages. The proportion presenting with gametocytes may vary according to background transmission levels. If gametocytes persist after treatment with ACTs, the effect on transmission in areas where a high proportion of cases present with gametocytes may prove suboptimal and justify the simultaneous use of primaquine.

Primaquine is more rapidly excreted than artesunate and this may account for the fact that the single dose of primaquine used in this study had a lower impact on gametocyte carriage than the artesunate regimens. The usefulness of primaquine as a gametocytocidal treatment may be improved by administering it after the ACT [14], [54].

For treatment policy to have a major impact on transmission several criteria need to be met: i) transmission is low to moderate; ii) the majority of people use public health rather than private facilities, or effective private sector interventions ensure adherence to policy; iii) public health facilities correctly prescribe the approved regimen, and iv) patients take the full course. These conditions are largely met in the refugee villages of Pakistan. The region is characterized by low endemicity and low immunity, well supported health care facilities are available in the Afghan refugee villages, and diagnosis of malaria to species level is maintained to a high standard [28]. The health facilities are well utilized by the Afghan refugees and Pakistanis living nearby. The effect of this policy is evident in the refugee populations: since 2005 falciparum malaria has virtually disappeared in all but a handful of the refugee villages [International Rescue Committee, Final Report, unpublished]. This reduction is attributed to prompt and accurate diagnosis and wider access to ACTs as prevention interventions were minimal.

The operational data from this region and elsewhere suggest that drugs showing faster gametocyte clearance in clinical trials do help to reduce transmission [20]–[23]. However, it is also important to note the results of recent research which suggest that drawing conclusions about the potential impact on transmission is not straightforward, for the following reasons:

- The effect of drugs on gametocyte carriage cannot be fully determined if only standard light microscopy (LM) is used. Research in the last decade shows that LM gives starkly different indications of gametocyte prevalence and densities compared to other techniques [55] and that sub-microscopic levels of gametocytes likely play an important role in transmission, perhaps particularly in low transmission settings [56].
- Gametocytes that are readily identified in blood following treatment may or may not be viable. Gametocytes may not be infective to mosquitoes either because they are recently emerged (naturally, gametocytes are not infectious to mosquitoes in the first 1–2 d of emergence [57]) or because the effect of the drug regimens have rendered them ‘sterile’ [58].
- It is unclear how the density of gametocytes in the blood links to potential for transmission success to blood feeding mosquitoes. Bousema & Drakeley [57] summarise the available data from membrane feeding studies showing that whilst there is an overall correlation, the variations within it are confusing; high density gametocytaemia can lead to minimal mosquito infection and very low densities lead to reasonable rates of infection.
- Even if solid data show that a particular drug does reduce the number of gametocytes which successfully infect, and lead to infectious, mosquitoes; the effect of this on malaria transmission cannot be stated with certainty. This effect would depend on what proportion of the gametocyte reservoir these patients would otherwise make up [56].

The comparative performance of artesunate and primaquine on gametocyte carriage in this study did not influence policy. The decision to use SP+AS was based on treatment efficacy and the need to protect against SP resistance through the use of an ACT combination. The fact that SP+AS led to reduced gametocyte carriage compared to the previous policy of chloroquine montherapy was reassuring, and raised the possibility of the regimen reducing transmission too.

SP+AS is now the first line treatment policy in Pakistan, Iran, Afghanistan and India. The continuing collection of molecular data on SP resistance in this region will be vital, given that the treatment efficacy of artesunate could mask the emergence of SP resistance by acting alone to provide a degree of clinical cure in the presence of a failing companion drug [59]. This risk could be reduced by introduction of a co-blister of SP plus artesunate.

## Supporting Information

Protocol S1 **Trial Protocol.**
(DOC)

Checklist S1 **CONSORT Checklist.**
(DOC)
