# Supplementary material for: Defining Plasmodium falciparum Treatment in South West Asia: A Randomized Trial Comparing Artesunate or Primaquine Combined with Chloroquine or SP
Source: PLoS One. 2012 Jan 31;7(1):e28957. doi: 10.1371/journal.pone.0028957 (PMC3269419; doi:10.1371/journal.pone.0028957)
Supplement: Protocol S1 — Trial Protocol. (DOC) [file pone.0028957.s001.doc]

**May 1999**

**LONDON SCHOOL OF HYGIENE & TROPICAL MEDICINE**

**Keppel Street, London WC1E 7HT**

Application Number ...........

(To be added by the Secretary) FORM A

**ETHICS COMMITTEE**

**APPLICATION TO CONDUCT A STUDY INVOLVING HUMAN SUBJECTS**

This form should be completed, signed by the Principal Investigator and Head of Department, and returned to Phoebe Roome, Personal Assistant to the Dean, LSHTM, Keppel Street, London WC1E 7HT.

Name of Principal Investigator ......Mark Rowland.....................................................

Appointment held ...Research fellow................... Date ..........1.11.99...........................

Other Personnel involved............Daniel Chandramohan..............................................

Title of project ........Studies on combination therapy with artesunate to delay the selection of resistance to antimalarial drugs in Pakistan...................................

..............................................................................................................

..............................................................................................................

I approve this project scientifically.

.............................................

(Signature of Head of Department)

Date ................................

Received by Ethics Committee

.............................................

.............................................

1. Give an outline of the proposed project. Sufficient detail of the protocol must be given to allow the Committee to make an informed decision. (Attach a summary of the project if this is more convenient).

Objectives:

To quantify the benefit of adding an artemisinin derivative to existing therapies with chloroquine and sulfadoxine-pyrimethamine in Pakistan. To compare this in randomized controlled trials against the currently recommended gametocytical and schizontocidal treatment of chloroquine plus primaquine. This will be assessed using the following indicators and end points: cure rate (elimination of parasitaemia without recrudescence), rate and time of parasite clearance, rate of resolution of fever, proportion of gametocyte carriers, transmissibility of gametocytes through mosquito feeding studies, tolerability, molecular characterisation of genetic diversity and resistance before and after treatment selection.

Rationale:

The increase in falciparum malaria in Pakistan over the last 15 years coincides with the spread of chloroquine resistance, which is now ubiquitous (Shah et al. 1997). We have reached the stage where chloroquine’s position as official first line treatment must be reconsidered. But in favour of what? Resistance to sulfadoxine-pyrimethamine (SP) stands at around 10% (Rowland et al. 1997). If this drug was substituted as first-line, selection for SP resistance might follow the same pattern observed in SE Asia where use of SP had to be abandoned after just a few years. The Pakistani public is losing faith with the national drug policy and with government services (just as Afghan refugees are with UNHCR services), and are turning to the unregulated private sector where a wide range of antimalarials are sold ad hoc. Treatment policy has to change to avert a resistance catastrophe.

It is conceivable that chloroquine might still have a useful role. We know that extended treatment with chloroquine can overcome some R1 infections in Pakistan (Rowland, unpubl.). A drug with stronger trophozoitocidal activity (i.e. artesunate) might bring about that extra reduction in parasite load that enables the companion drug (chloroquine) to eliminate an otherwise R1 infection. We therefore propose to compare CQ (day 1,2,3) + AS (1,2,3) vs CQ (day 1,2,3).

If this combination fails it may be too late to preserve chloroquine in Pakistan (except for vivax treatment). Then it becomes even more crucial to preserve SP. The second comparison is therefore between SP (day 1) + placebo vs SP (day 1) + AS (day 1).

The national policy of Pakistan is to co-treat with primaquine during chloroquine or SP therapy of falciparum cases to eliminate gametocytes and thereby block transmission. Little research is being done with primaquine, yet it is still considered a useful drug for eradicating gametocytes that persist after treatment with purely schizontocidal drugs. Primaquine might, conceivably, slow down the selection of resistance by killing off resistant gametocytes that arise from selection within the patient by schizontocidal drugs. More research on primaquine needs to be done before a decision can be taken by Pakistan’s MOH to abandon it in favour of artesunate. Primaquine is conventionally administered on the first day of chloroquine treatment in Pakistan. Since it has a half life of only 6h and is eliminated from the body in 1 day (WHO, 1984), it may be more efficacious to administer primaquine on the 3^rd^ day of chloroquine treatment. We therefore propose to compare the gametocytocidal efficacy of day 1 and day 3 primaquine treatments.

Artesunate was once thought to have gametocytocidal activity; this may now be in doubt (Targett pers. comm.). It would, nevertheless, be valuable to compare AS treatment vs primaquine treatment vs placebo treatment (all administered on the 1st day of chloroquine treatment of clinical cases), and to monitor trophozoite and gametocyte carriage from thereon. Even if the 1-day AS treatment failed to kill gametocytes and reduce gametocyte load directly it should achieve this effect indirectly through a reduction of trophozoite load. The effect of 1-day AS treatment on trophozoites might even enable chloroquine to effect a full clinical cure of an otherwise chloroquine-R1 infection (and thus the “1-day AS : 3-day CQ” regimen must be compared with the “3-day AS : 3-day CQ” regimen described earlier to determine which of the two doses of AS is optimal).

Recent evidence from the Gambia (Targett, pers.comm.) and from Pakistan (Rowland, unpublished) indicates that treatment of clinical infections with SP stimulates gametocyte production over the next 10 days even though asexual stages are quickly eliminated from the peripheral circulation. These gametocytes in the peripheral circulation may be fully viable, and the phenomenon may explain why SP resistance was selected so quickly in SE Asia when SP became 1^st^ line drug. This is an argument for always combining SP with a second antimalarial. If this second drug were artesunate it should prevent gametocyte production by its rapid action on asexual stages. If the second drug was primaquine it might have the same effect but by a different mechanism i.e. by killing the gametocytes “stimulated” by SP treatment. It is therefore proposed to include an SP+primaquine arm in the SP+AS vs SP+placebo comparison.

To determine the viability and transmissibility of any gametocytes (and also to detect sub-patent gametocytaemias) still present after treatment it is also proposed to carry out mosquito feeding studies directly on patients >13 years of age on the 7^th^ day after the start of combination therapy with either CQ, CQ+AS, CQ+PRIM., SP, SP+AS, SP+PRIM and to incubate any midgut infections to the oocyst stage. To determine the genetic consequences of any selection from the different drugs (i.e. CQ, AS, or primaquine), the mosquito midgut infections would be preserved for further genetic studies in UK, as would blood samples taken from initial and recrudescent infections. The genetic studies in LSHTM would be funded from other sources.

Detection of the *cg2* genotype is an useful epidemiological tool for assessing the distribution of chloroquine resistance (Adagy and Warhust, 1999). It is now believed that *pf Tcr* gene located close to *cg2* in chromosome 7 is linked with chloroquine resistance and methods are available for detecting *pfTcr* (Wellems T.E, personal communication). Mutations in *dhfr* and *dhps* genes have been associated with resistance to pyrimethamine and suphadoxine respectively. Simple and rapid tests have been developed to detect these polymorphisms, using PCR followed by restriction digestion (Duraisingh et al. 1998).

To improve our understanding of the genetic basis of drug resistance we will genotype parasites from blood samples of patients with treatment failure in this study. Blood samples of 20 patients from each arm of the study who had parasitological treatment failure will be selected randomly, together with midgut infections, and analysed for genetic markers of resistance to chloroquine and sulphadoxine/pyrimethamine.

4.2 Experimental design and methods:

Protocol design:

[[1]](#footnote-2)4bRandomised, double-blind, placebo control trial comparing (1) artesunate (AS) and chloroquine (CQ), vs CQ alone, vs CQ and primaquine (PR); (2) AS and sulphadoxine-pyrimethamine (S/P), vs S/P alone, vs S/P and PR.

Patients will be randomly assigned to one of the following treatment groups:

Adizai refugee camp in NWFP

- CQ (day1,2,3) + placebo (day 1, 3) vs
- CQ (day 1,2,3) + PR (day 1) + placebo (day 3) vs
- CQ (day 1,2,3) + PR (day 3) + placebo (day 1) vs
- CQ (day 1,2,3) + AS (day 1) + placebo (day 3)

Azakhel refugee camp in NWFP

- S/P (day 1) + placebo (day 1) vs
- S/P (day 1) + AS (day 1) vs
- S/P (day 1) + PR (day 1)

Sindh rural health centre

- CQ (day 1,2,3) + placebo (day 1,2,3) vs
- CQ (day 1,2,3) + AS (day 1,2,3)

Study sites - two areas with high malaria prevalence: Afghan refugee settlements in NWFP (Adizai and Azakhel) and Pakistani communities in Sindh province (Noshero Feroze).

Entry Criteria: adults or children > 5 yrs, weight > 5 kg, monoinfection with *P.faliciparum*, history of recent fever, consent from patient or parent. Exclusion: patients with signs of severe malaria.

Sample size calculation

Chloroquine combinations:

100 in each of the 6 study arms (assumes 5 % loss to follow up) = 400 cases will be recruited in NWFP (4 arms) and 200 in Sindh (2 arms). Assumptions for sample size (confidence level 95%, power 80%): 1. Estimated frequency of recrudescence in the chloroquine group: 30%, expected recrudescence in the test combination group: 10% or less. 2. Estimated prevalence of gametocyte positives after 7 days in the chloroquine group: 50%; in the test combination group: 25%.

S/P combinations:

150 in each of the 3 study arms = 450 cases will be recruited in NWFP. Assumptions for sample size (confidence level 95%, power 80%): 1. Estimated frequency of recrudescence in the S/P group: 10%, expected recrudescence in the test combination group: 1%. 2. Estimated prevalence of gametocyte positives after 7 days in the S/P group: 50%; in the test combination group: 25%.

Drugs and treatment regimens:

- AS dispensed as 50mg tablets at oral dose: 4mg/kg/day
- S/P dispensed orally as tablets (500 mg sulphadoxine and 25mg pyrimethamine)at oral dose: 25mg/kg based on sulphadoxine component, given once only
- CQ dispensed as 150mg base tablets at oral dose of base: 10mg/kg/day given once on day 0 & day 1, and 5mg/kg once on day 2.
- Primaquine will be dispensed as 7.5mg tablets at oral dose: 0.5mg/kg, given once only. All cases receiving primaquine will be screened for G6PD. Pregnant women excluded

Protocol operation:

Patients will be recruited by passive case detection from selected primary health care centres where diagnostic facilities or quality control are provided by HNI. In NWFP, two ARCs at Adizai (population 3,950) and Azakhel (population 16,248) in Peshawar and Nowshera districts will be the principal experimental sites. Each family is registered at the clinic which maintains family records. In Sindh, along the River Indus, a rural health centre in the most heavily affected district (Noshero Feroze) has been selected. This is presently being upgraded by HNI as part of our insecticide treated net project in the area. The catchment population is 30,000.

Subjects fulfilling the entry criteria will be medically screened: history, physical examination, malaria smear, and blood for Hb and/or PCV and white cell count. The 250 randomly-selected cases who will [[2]](#footnote-3)4creceive primaquine treatment will be screened for G6PD deficiency at admission, and cases positive for this condition will be closely observed over the next 48h for any adverse reaction i.e. haematuria. The recommended single dose of 0.5mg/kg primaquine is well tolerated (WHO, 1990) and it is standard practice to administer higher doses (primaquine for 5 days) for vivax treatment in Pakistan: no haemolytic reaction has been observed in G6PD deficient Afghans in previous studies (Rowland & Durrani, 1999).

Patients will be allocated in equal numbers to each regimen using a supplied random number chart. Every antimalarial treatment will be administered by HNI health workers under supervision of a HNI licensed physician. The dose will be determined using supplied weight dosing charts. The same dose will be given again if vomiting occurs in first hour. Patients will return to the clinic 24 and 48 hours later for drug administration and for scheduled tests at 72 hours, days 7, 14, 21, 28, (and 42 for S/P arms). If a patient fails to report fieldworkers will locate him/her at his/her home address.

Follow up blood samples for haematology (Hb, PCV and WBC count) will be done using two different schedules: first 20% (see chant below): on days 0, 7 & 28. Remaining subjects: haematology on days 0 & 28.

| **Day** | **0** | **1** | **2** | **3** | **4** | **5** | **6** | **7** |  | **14** | **21** | **28** | **42** |
| --- | --- | --- | --- | --- | --- | --- | --- | --- | --- | --- | --- | --- | --- |
| History | X |  |  |  |  |  |  | X |  | X | X | X | X |
| Examination (clinical) | X | X | X | X^1^ |  |  |  | X |  | X | X | X | X |
| Temperature | X | X | X | X^1^ |  |  |  | X |  | X | X | X | X |
| Blood film | X | X | X | X^1^ |  |  |  | X |  | X | X | X | X |
| Filter paper PCR^2^ | X |  |  |  |  |  |  | X |  | X | X | X | X |
| Filter paper PK^2^ | X | X | X | X |  |  |  | X |  | X | X | X | X |
| Haematology | X |  |  |  |  |  |  | X |  |  |  | X | X |
| Thick & thin smears | X | X | X | X |  |  |  | X |  | X | X | X | X |

PCR/PK^2^ = A sample for PCR and for PK will also be obtained if the patient reports with clinical symptoms and a positive blood film at any time other than the above.

Thick and thin blood smears will be taken on days 0, 1, 2, 3, 7, 14, 21, 28, 42 (for S/P). Enrolment requires 5000 parasites/mm^3^.

A random selection of 20 patients (aged >13y) from each arm of the two studies in NWFP will be asked to volunteer for mosquito feeding studies on day 7 after treatment. The purpose of the study will be explained and written informed consent obtained. It is also hoped that up to 20 subjects (aged>20y) with recrudescent infections from each arm will also volunteer for feeding studies. Subjects will exposed to 30 unfed colony-mosquitoes (A.stephensi) reared in our insectary at Adizai ARC. Mosquitoes from single feeds will be kept in separate cages for 7 days and then dissected, oocysts counted, and midguts preserved for molecular genotyping at LSHTM using methods of Adagy and Warhust (1999) and Duraisingh et al. (1998).

| Activity | **Study Period (Months) Jul 2000 to Apr 2001** | | | | | | | | | | |
| --- | --- | --- | --- | --- | --- | --- | --- | --- | --- | --- | --- |
|  | **1** | | **2** | **3** | **4** | **5** | **6** | **8** | **7** | **9** | **10** |
| Initial planning and staff recruitment |  |  |  |  |  |  |  |  |  |  |  |
| Training of staff |  |  |  |  |  |  |  |  |  |  |  |
| Drug Trials |  |  |  |  |  |  |  |  |  |  |  |
| Data entry |  | |  |  |  |  |  |  |  |  |  |
| Data Analysis |  | |  |  |  |  |  |  |  |  |  |
| External coordinators/clinical monitors visits |  | |  |  |  |  |  |  |  |  |  |
| Preparation and submission of report |  | |  |  |  |  |  |  |  |  |  |

Reasons for discontinuation: severe malaria, as per the WHO definition, day 3 parasite count >=25%, parasitaemia present on day 7, recurrent parasitaemia, patient’s decision, consumption of a drug with antimalarial activity, haemolytic response to primaquine treatment.

Appropriate statistics will be used to analyse the results, as described in the full project document.

[[3]](#footnote-4)4d4.3 Ethical considerations

This clinical study will be conducted in accordance with the principles laid down by the World Health Assembly of 1975 on Ethics in Human Experimentation and the Helsinki Declaration. The study will adhere to the standards established for Good Clinical Practices (GCP) and conform to the TDR Standard Operating Procedures (SOP). The protocol of this study will be approved before it is initiated by LSHTM Ethical Committee. Written approval to perform this trial is being obtained from the Pakistan Medical Research Council.

If the field research is successful, genetic studies will be undertaken on the samples taken, and it would be hoped the project may be expanded in following years to cover other refugee camps to monitor any selection of drug resistance.

4.4 Critical assessment and limitations

No identifiable limitations. Assessment similar to studies on AS proposed for other regions.

References

Adagu IS, Warhurst DC (1999). Association of cg2 and pfmdr1 genotype with chloroquine resistance in field samples of plasmodium falciparum from Nigeria. Parasitology 119, 343-348.

Duraisingh MT, Curtis J, Warhurst DC (1998). Plasmodium facliparum: detection of polymorphisms in the dihydrofolate reductase and dihydropteorate synthatase genes by PCR and restriction digestion. Experimental parasitology 89, 1-8.

M Rowland, N Durrani. 2000. Randomized controlled trials of 5- and 14-days primaquine therapy for radical cure of vivax malaria. *Transactions of the Royal Society of Tropical Medicine & Hygiene* 93, 641-643.

Rowland,M., N Durrani, S Hewitt, E Sondorp. 1997b. Resistance to falciparum malaria to chloroquine and sulphadoxine-pyrimethamine in Afghan refugee settlements in western Pakistan: surveys by general health services using a simplified in-vivo test. *Tropical Medicine and International Health*, 11(2), 1049-1056

I Shah, M Rowland, P Mehmood, C Mujahid, F Raziq, S Hewitt, N Durrani. 1997. Chloroquine resistance in Pakistan and the upsurge of falciparum malaria in Pakistani and Afghan populations. *Annals of Tropical Medicine and Parasitology*, 91, 591-602.

WHO. 1984. Advances in malaria chemotherapy. Technical report series 711.

WHO. 1990 Practical chemotherapy of malaria. Technical report series 805.

2. State the intended value of the project. (If this project or a similar one has been done before what is the value of repeating it?).

This is one of a number of multicentre trials investigating the efficacy and feasibility of combination therapy and its clinical and parasitological effects, and the effect on the rate of selection of resistance. The pattern of resistance and immune status in S.Asia is unique and the study cannot be considered a repetition of studies undertaken elsewhere.

3. Specify the number, age, sex, source and method of recruiting subjects for the study. Attach a copy of any advertisement to be used.

Malaria patients will be recruited by passive case detection from primary health care centres where diagnostic facilities or quality control are provided by HNI for Afghan refugees. About 1000 cases aged > 5y will be recruited with written consent and allocated at random to one of the nine treatment arms.

4. State the likely duration of the project, and where it will be undertaken.

Will run from June 2000-April 2001 in the camps of Adizai and Azakhel in NWFP, and at a rural health centre in Sindh province, Pakistan.

5. Specify the procedures (including interviews) involving human subjects.

Subjects fulfilling the entry criteria will be medically screened: history, physical examination, malaria smear, and blood for Hb and/or PCV and white cell count. About 250 randomly-selected cases due to receive primaquine treatment will be screened for G6PD deficiency at admission. Every antimalarial treatment will be administered by HNI health workers under supervision of a HNI licensed physician using standard doses determined using weight dosing charts. Blood smears and samples for haematology (Hb, PCV and WBC count) will be done at intervals. Sample will be taken if the patient reports with clinical symptoms and a positive blood film at any time other than the above; radical treatment will be administered.

A random selection of 20 patients from each arm in NWFP will be asked to volunteer for mosquito feeding studies on day 7 after treatment. The purpose will be explained and written informed consent obtained. Subjects will exposed to 30 unfed colony-mosquitoes (A.stephensi) reared in our insectary at Adizai ARC.

6. State the potential hazards, if any, and the precautions being taken to meet them (include information on hazardous substances that will be used or produced, and the steps being taken to reduce risks).

The 250 cases due to receive primaquine treatment will be screened for G6PD deficiency at admission, and cases positive for this condition will be closely observed over the next 48h for any adverse reaction i.e. haematuria. Furthermore the recommended single dose of 0.5mg/kg primaquine is routinely administered without this attention and is well tolerated (WHO, 1990). It is standard practice to administer higher doses (primaquine for 5 days) for vivax treatment in Pakistan, and no haemolytic reaction has been observed in G6PD deficient Afghans in previous studies (Rowland & Durrani, 1999).

7. State the procedures or activities which may cause discomfort or distress and the degree of discomfort or distress likely to be entailed by the subjects.

Taking of repeated blood smears may cause little discomfort. However results of these examinations will be of clinical benefit for the subjects. Venepuncture causes distress to a few children. With some of these it may be necessary to abandon the procedure is the level of disctress is progressively heightened.

8. Specify the degree of confidentiality to be maintained with respect to the data collected and the method of achieving this.

Data will be held by medical staff at the clinics and will be treated with the same degree of confidentiality as all medical records.

9. State the personal experience of the applicant and of senior collaborators in the study in the field concerned.

The PI and his local collaborators have run clinical RCT trials on malaria chemotherapy in Afghan camps on several occasions in the past and have published the findings in international journals.

10. State the manner in which consent will be obtained (eg verbal, written, witnessed) and supply copies of the information sheet and consent form. Healthy volunteers and patients will require different information sheets and consent forms. (See notes attached at end of this form).

Verbal consent and witnessed consent is obtained after explanation by the clinic health worker in the local language. Whenever possible written consent will be obtained from the patient or his parent/guardian. Information and consent forms are attached.

11. State what medical supervision is available and its location in relation to the subjects.

Each camp and RHC has fully qualified and licensed HNI or government physicians to provide supervision and treatment, and competent technicians to undertake microscopical diagnosis.

12. Is the study initiated/sponsored by a pharmaceutical or other industrial company?

NO by WHO/TDR

13. (a) Does the project involve pre-marketing use of a drug/appliance or a new use for a marketed product?

NO

(c) In a study on healthy volunteers does the company agree to abide by the current guidelines of the ABPI for healthy volunteers?

YES / NO

If YES a copy of the proposed volunteer contract should be attached.

(d) What is the regulatory status of the drug under the Medicines Act 1968: Product Licence / Clinical Trial Certificate (CTC) / Clinical Trial Exemption (CTX) / Doctor or Dentist Exemption (DDX)? If CTC, CTX or DDX a copy of the certificate should be attached.

14. Will payments be made to subjects?

YES

A flat rate of 50 rupee (half day salary) for each feeding study (30 mosquitoes per patient).

15. Will the level of service or support available to study subjects be lower after the study than during the study?

NO

16. Describe the measures to be taken to communicate the results of the study to study subjects, their representatives, local government, national government and other relevant bodies who could use the results of the study to improve the lives of the study subjects.

The clinic staff and PHC workers will disseminate the results to the refugee communities. Depending on the outcome of treatment national guidelines may be revised in discussion with UNHCR and Pakistan Medical Research Council (both of whom are regularly reported to by the HNI control project). Data and findings will be submitted to TDR and published by the PI and colleagues.

17. Include any other relevant information.

This research protocol complies with the principles of the Declaration of Helsinki, amended as stated in Section II, item 15.1 of TDR/Form/99 (collaborative research project.

18. Where the research is to take place overseas, the Principal Investigator **must** seek ethical approval, through his/her overseas collaborators, in the country(s) concerned. Approval will not be granted by the LSHTM Ethics Committee until this written approval is submitted.

Please list the countries where research is being undertaken

 Other countries .....Pakistan.......................

UK only (Please list) ................................................

....................................................................

....................................................................

....................................................................

....................................................................

Please submit formal ethical approval statement given by local committee within each country. If ethical approval has not yet been obtained from a local committee in the country, indicate to whom the proposal has been submitted and when a response is expected.

....................................................................................................................................

....................................................................................................................................

Signature of applicant

Medically qualified NO

Other qualifications (please state) PhD entomology and parasitology

Are you a member of a medical protection organisation? NO

Are you a member of any other protection organisation? NO

NB My co-investigator at LSHTM, Daniel Chandramohan, does fulfil the above medical qualification requirements.

# CONSENT FORM for the drug trial

**Title**: Efficacy of combinations of antimalarial drugs

**Investigators**: Mark Rowland, Abdur Rab

**Patient Interviewers:** Dr Rab, Dr Fayaz, Mr Naeem, Mr Muzafa Khan

You may be aware that malaria is a major problem in Pakistan and Afghanistan and that treatment with chloroquine or Fansidar sometimes fails to cure malaria. One way of preventing treatment failure is to treat with combination of antimalarial drugs. Combinations of a new drug called artesunate with the currently used drugs namely chloroquine, primaquine, or Fansidar may prevent developing resistance to these drugs. Studies done in Thailand have shown that these combinations are safe. However, we do not know whether these combinations of drugs are useful in Pakistan and we believe this information is very important for treatment and control of malaria in this region. So we need your help to evaluate the usefulness and safety of several combinations of drugs for the treatment of malaria.

If you agree to take part in this study this is what will happen. By lottery you will be allocated to one of the nine groups of treatments that are being evaluated. Some groups will receive one of the new treatments which stand to be better while others receive the current treatment. The quality of treatment stands to be at least as good as what you would normally receive. We will ask you to come the clinic on day 1,2,3,7,14,21,28, and 42 for check up and on these days we will take a small amount of blood to examine for malaria. If you fail to visit the clinic a field worker will visit your house for taking blood. The study will finish on day 42.

All information collected form you will not be disclosed to anyone by the project staff. Participation in this trial is voluntary. If you do not wish to participate in this study, you are free to do so. You may also withdraw from the study at any point during the follow up period. Non participation in the study will not affect the health care you receive now or in the future.

Do you understand what I have said? Do you have any questions? Would you like to participate in the study

I certify that I have explained the above to ________________________________, that she/he understood what I said and she/he agreed to participate in the study.

Signature …………………………… Date …………….

[Name]

I have understood the explanation given to me by __________________________ and I agree to join the study.

Signature or Mark ……………………………….. Date

# [Name]

# CONSENT FORM for the mosquito feeding study

**Title**: Efficacy of combinations of antimalarial drugs

**Investigators**: Mark Rowland, Abdur Rab

Thank you very much for taking for in our study that is evaluating the usefulness of combination of drugs for treating malaria. You may recall that we explained at the time you agreed to take part in this study that combinations of a new drug called artesunate with the currently used drugs namely chloroquine or Fansidar may prevent developing resistance to these drugs. Shortly after treatment with chloroquine and Fansidar, if mosquitoes bite the patient then the mosquitoes can become infected with the malaria parasite and give the disease to other people they bite. We want to find out if use of artesunate or primaquine will prevent mosquitoes becoming infected. In order to understand this effect we have to assess whether mosquitoes will be infected from people who have been treated with these artesunate or primaquine. We need your help to study this.

If you agree to take part in this study this is what will happen. Seven days after treatment a paper cup with mosquito inside will be placed on your arm and the mosquitoes will feed on you. These mosquitoes are reared in out insectory and so we are certain that they do not carry any germs.

All information collected form you will not be disclosed to anyone by the project staff. Participation in this trial is voluntary. If you do not wish to participate in this study, you are free to do so. You may also withdraw from the study at any point. Non participation in the study will not affect the health care you receive now or in the future.

Do you understand what I have said? Do you have any questions? Would you like to participate in the study?

I certify that I have explained the above to ________________________________, that she/he understood what I said and she/he agreed to participate in the study.

Signature …………………………… Date …………….

[Name]

I have understood the explanation given to me by __________________________ an I agree to join the study.

Signature or Mark ……………………………….. Date

[Name]

1. ^4b^ [↑](#footnote-ref-2)
2. ^4c^ [↑](#footnote-ref-3)
3. ^4d^ [↑](#footnote-ref-4)
